# Is Night Surgery a Nightmare for Lung Transplantation?

**DOI:** 10.3389/ti.2024.12816

**Published:** 2024-07-02

**Authors:** Sébastien Tanaka, Christian De Tymowski, Erevan Dupuis, Alexy Tran-Dinh, Brice Lortat-Jacob, Adela Harpan, Sylvain Jean-Baptiste, Sandrine Boudinet, Chahra-Zad Tahri, Mathilde Salpin, Yves Castier, Pierre Mordant, Hervé Mal, Antoine Girault, Enora Atchade, Philippe Montravers, the Bichat Lung Transplant Group

**Affiliations:** ^1^ Assistance Publique - Hôpitaux de Paris (AP-HP), Department of Anesthesiology and Critical Care Medicine, Bichat-Claude Bernard Hospital, Paris, France; ^2^ Réunion Island University, French Institute of Health and Medical Research (INSERM), U1188 Diabetes Atherothrombosis Réunion Indian Ocean (DéTROI), CYROI Platform, Saint-Pierre, France; ^3^ French Institute of Health and Medical Research (INSERM) U1149, Center for Research on Inflammation, Paris, France; ^4^ Université Paris Cité, Paris, France; ^5^ French Institute of Health and Medical Research (INSERM) U1148, Laboratory for Vascular Translational Science, Paris, France; ^6^ Assistance Publique - Hôpitaux de Paris (AP-HP), Department of Pneumology and Lung Transplantation, Bichat-Claude Bernard Hospital, Paris, France; ^7^ Assistance Publique - Hôpitaux de Paris (AP-HP), Department of Vascular and Thoracic Surgery, Bichat-Claude Bernard Hospital, Paris, France; ^8^ PHERE, Physiopathology and Epidemiology of Respiratory Diseases, French Institute of Health and Medical Research (INSERM) U1152, Paris, France

**Keywords:** mortality, lung transplantation, outcome, morbidity, operative time

## Abstract

Night work is frequently associated with sleep deprivation and is associated with greater surgical and medical complications. Lung transplantation (LT) is carried out both at night and during the day and involves many medical healthcare workers. The goal of the study was to compare morbidity and mortality between LT recipients according to LT operative time. We performed a retrospective, observational, single-center study. When the procedure started between 6 AM and 6 PM, the patient was allocated to the Daytime group. If the procedure started between 6 PM and 6 AM, the patient was allocated to the Nighttime group. Between January 2015 and December 2020, 253 patients were included. A total of 168 (66%) patients were classified into the Day group, and 85 (34%) patients were classified into the Night group. Lung Donors’ general characteristics were similar between the groups. The 90-day and one-year mortality rates were similar between the groups (90-days: n = 13 (15%) vs. n = 26 (15%), *p* = 0.970; 1 year: n = 18 (21%) vs. n = 42 (25%), *p* = 0.499). Daytime LT was associated with more one-year airway dehiscence (n = 36 (21%) vs. n = 6 (7.1%), *p* = 0.004). In conclusion, among patients who underwent LT, there was no significant association between operative time and survival.

## Graphical Abstract

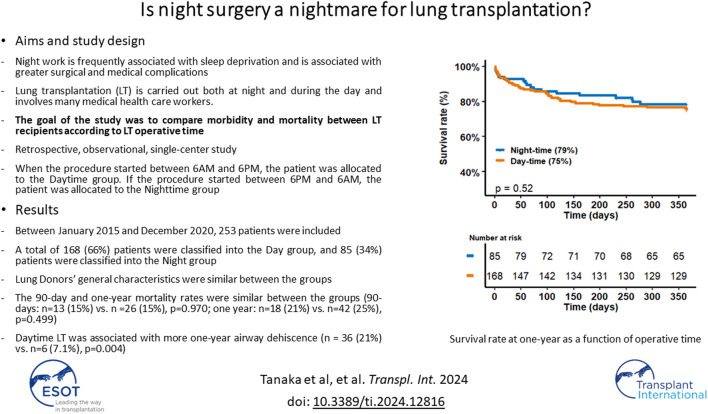

## Introduction

Sleep deprivation is a major public health issue that concerns the entire population but also healthcare professionals. Sleep deprivation is known to have major consequences for attention and medical reasoning and can lead to serious medical errors [1–3]. Night work is frequently associated with sleep deprivation and circadian disruption and is also associated with greater surgical and medical complications [4–7].

For more than 30 years, lung transplantation (LT) procedures have been performed worldwide; these procedures involve both night and day care and involve many medical and nonmedical healthcare workers, especially surgeons, anesthesiologists and intensivists [8, 9]. A large retrospective study in North America including more than 27,000 lung and heart transplant recipients did not reveal any difference in survival according to the procedure schedule (night, 7 PM-7 AM versus day, 7 AM-7 PM) [10]. Interestingly, among lung transplant recipients, there was a slightly greater rate of airway dehiscence associated with nighttime transplants.

Given that transplantation organizational procedures and caregivers working time legislation differ from one country to another [11, 12], the goal of the present study was to compare lung transplant characteristics and outcomes performed in a French LT center according to the operative time.

## Materials and Methods

### Study Population

All consecutive patients who underwent LT at Bichat-Claude Bernard Hospital, Paris, France, from January 2015 to December 2020 were retrospectively included in this observational, single-center analysis. The data were collected prospectively. The Paris North Hospital Institutional Review Board (Paris Diderot University, Assistance Publique Hôpitaux de Paris No. 0007477) reviewed and approved the study.

## Objectives

- The main objective of this study was to compare one-year mortality between LT recipients according to LT operative time.
- The secondary objective was to assess the associations between operative time and donor characteristics, recipient general characteristics, perioperative data, postoperative outcomes and complications.

### Operative Time

Total operative time was defined as the complete time from arrival to discharge from the operating room, including the time of anesthetic management and surgical procedure.

Patients were stratified by operative time. When the procedure started between 6 AM and 6 PM, the patient was allocated to the daytime group. If the procedure started between 6 PM and 6 AM, the patient was allocated to the nighttime group.

### Perioperative Management

Perioperative care was standardized for all patients according to current practices [13–15]. After the surgical procedure, all patients were admitted to our surgical intensive care unit (ICU). Postoperative ECMO was required in case of PGD3, severe pulmonary arterial hypertension, perioperative cardiac dysfunction and in the case of ARDS in the context of early pneumonia. ECMO was also required if intraoperative bleeding and transfusion have been consistent and could lead to pulmonary edema and ARDS.

The LT team was composed of a senior anaesthetist and a resident, and the surgical staff of a senior surgeon, a junior surgeon and a resident. There were five senior surgeons with at least 5 years’ experience as junior surgeons.

### Data Collection

- General demographic data of the donors, including age, sex, cigarette use, best PaO_2_/FiO ratio and length of mechanical ventilation, were collected.
- Demographic characteristics, underlying disease, and medication use during the pretransplant assessment period were prospectively recorded.
- Perioperative data, including length of total operative time, duration of surgery, procedure start time (i.e., arrival in the operating room), need for transfusion and need for ECMO support, were collected.
- Postoperative complications and variables during ICU hospitalization following LT were also recorded, such as postoperative ECMO support, primary graft dysfunction (PGD) defined according to the ISHLT revised definition [16], acute kidney injury, need for renal replacement therapy, episode of pneumonia during the ICU stay, duration of vasopressor agent administration, duration of mechanical ventilation and length of stay in the ICU. Airway dehiscence, acute cellular and humoral rejection during the first postoperative year, and mortality at 90 days and 1 year were also prospectively collected.

### Statistical Analysis

Continuous variables are expressed as medians with interquartile ranges (IQRs) and were compared with the Mann‒Whitney *U* test. Categorical variables are expressed as counts and percentages and were compared with Fisher’s exact test or the chi-square test, as appropriate. Time-to-event analyses were estimated with Kaplan-Meier analyses, and survival differences were analyzed using a log rank test. Multivariate associations were computed with binary logistic regression models. For all the models, variables with nominal 2-tailed *p* values less than 0.1 were entered into the multivariate model, except for variables with obvious collinearity. All the statistical analyses were performed using R statistical software^1^. And RStudio (version 1.3.1056, ^©^ 2009–2020 RStudio, PBC). A *p*-value <0.05 was considered to indicate statistical significance.

## Results

### Population

Between January 2015 and December 2020, 269 patients underwent LT at our institution. Patients who underwent liver and lung transplantation were excluded from the analysis (n = 3). Thirteen patients were excluded from the analysis because perioperative data were incomplete. A total of 253 patients were ultimately included in this study.

A total of 168 (66%) patients were classified into the Day group, and 85 (34%) patients were classified into the Night group.

The median LT procedure time was 440 [390, 530] minutes, and the median surgical time was 320 [270, 390] minutes. The median procedure start times were 10 am [8 am–12 am] and 2 am [10 pm-4 am] for patients in the day and night groups, respectively.

The general characteristics of the patients in the whole population and according to the LT schedule are presented in Table 1. Interestingly, LT in COPD/emphysema patients were statistically more often performed at night (Nighttime, n = 38 (45%) vs. Daytime, n = 53 (32%), *p* = 0.039).

**TABLE 1:** General characteristics of the patients in the whole population and in the different groups.

General characteristics	Overall population (n = 253)	Night (n = 85)	Day (n = 168)	*p*
Age, years, median [IQR]	57 [50–62]	57 [51–63]	57 [50–62]	0.525
Male sex, *n* (%)	162 (64)	55 (65)	107 (64)	0.874
BMI (kg/m^2^), median [IQR]	24.0 [20.0–27.0]	24.6 [20.0–27.0]	24.0 [20.0–27.0]	0.699
Diabetes mellitus, n (%)	26 (10)	11 (13)	15 (8.9)	0.321
Chronic coronary disease n (%)	10 (4.0)	3 (3.5)	7 (4.2)	>0.999
Mean pulmonary artery pressure (mmHg), median [IQR]	25 [20–30]	24 [20–28]	26 [21–30]	0.146
Need of preoperative ECMO, n (%)	19 (7.5)	9 (11)	10 (6.0)	0.186
Diagnosis leading to LT				
COPD/emphysema, n (%)	91 (36)	38 (45)	53 (32)	0.039
Pulmonary fibrosis, n (%)	122 (48)	34 (40)	88 (52)	0.063
Other pathologies, n (%)	41 (16)	13 (15)	28 (17)	0.780
Double LT, n (%)	173 (68)	57 (67)	116 (69)	0.748

Continuous variables are expressed as medians and interquartile ranges (IQRs) and were compared using the Mann‒Whitney *U* test. Categorical variables are expressed as n (%) and were compared with Fisher’s exact test. BMI, body mass index; COPD, chronic obstructive pulmonary disease; LT, lung transplantation.

### Donors’ Characteristics

The donors’ characteristics were similar between the two groups and are described in Supplementary Table S1.

### Pre- and Postoperative Variables

The length of total operative time was statistically longer during the day than at night (7h40 [6h40-8h50] vs. 6h50 [5h50-8h30], *p* = 0.002).

The distribution of surgeries was fairly homogeneous between surgeons. With the exception of one surgeon, all surgeons performed the majority of their grafts during the day, and their proportion of daytime grafts was identical to their proportion of night-time grafts (Supplementary Figure S2).

Interestingly, airway dehiscence was more frequently associated with daytime transplantations (n = 36 (21%) vs. n = 6 (7.1%), *p* = 0.004). The pre- and postoperative variables are expressed in Table 2.

**TABLE 2:** Pre- and postoperative variables.

Per/postoperative and outcome variables	Overall population (n = 253)	Night (n = 85)	Day (n = 168)	*p*
length of total operative time, hours, median [IQR]	7h20 [6h30-8h50]	6h50 [5h50-8h30]	7h40 [6h40-8h50]	0.002
Length of surgery, hours, median [IQR]	5h20 [4h30-6h30]	5h12 [4h20-6h30]	5h30 [4h30-6h30]	0.414
Peroperative transfusion ≥ 2RBC, n (%)	122 (48)	43 (51)	79 (47)	0.592
Need of peroperative ECMO, n (%)	178 (70)	57 (67)	121 (72)	0.414
SAPSII score on ICU admission, median [IQR]	43 [38, 52]	43 [40, 48]	45 [38, 54]	0.491
SOFA on ICU admission, median [IQR]	7.0 [6.0–9.0]	7.0 [5.0–9.0]	7.0 [6.0–9.0]	0.170
Duration of vasopressor agent administration, days, median [IQR]	2.0 [1.0–4.0]	2.0 [1.0–4.0]	2.0 [1.0–4.0]	0.702
Need of ECMO support during ICU stay, n (%)	73 (29)	24 (28)	49 (29)	0.877
Stage III PGD, n (%)	48 (19)	16 (19)	32 (19)	0.966
Acute kidney injury, KDIGO stage 3, n (%)	36 (14)	11 (13)	25 (15)	0.677
Renal replacement therapy, n (%)	29 (11)	8 (9.4)	21 (12)	0.466
Postoperative pneumonia, n (%)	219 (87)	75 (88)	144 (86)	0.579
Duration of MV, days, median [IQR]	3 [1, 20]	3 [1, 11]	4 [1, 21]	0.405
ICU length of stay, median [IQR]	17 [10–33]	16 [11–28]	17 [10–33]	0.618
Acute humoral rejection during the first year, n (%)	44 (18)	15 (18)	29 (18)	0.957
Acute cellular rejection during the first year, n (%)	49 (20)	18 (22)	31 (19)	0.653
Airway dehiscence during the first year, n (%)	42 (17)	6 (7.1)	36 (21)	0.004
90-days mortality, n (%)	39 (15)	13 (15)	26 (15)	0.970
One-year mortality, n (%)	60 (24)	18 (21)	42 (25)	0.499

Continuous variables are expressed as medians and interquartile ranges (IQRs) and were compared using the Mann‒Whitney *U* test. Categorical variables are expressed as n (%) and were compared with Fisher’s exact test. ECMO, extracorporeal membrane oxygenation; ICU, intensive care unit; KDIGO, kidney disease-improving global outcomes; MV, mechanical ventilation; PGD, primary graft dysfunction; RBC, red blood cell; SAPS-II, Simplified Acute Physiology Score II; SOFA, sepsis-related organ failure assessment; ICU, intensive care unit.

### Mortality at 90 Days and One Year According to Day or Night LT

**FIGURE 1:**
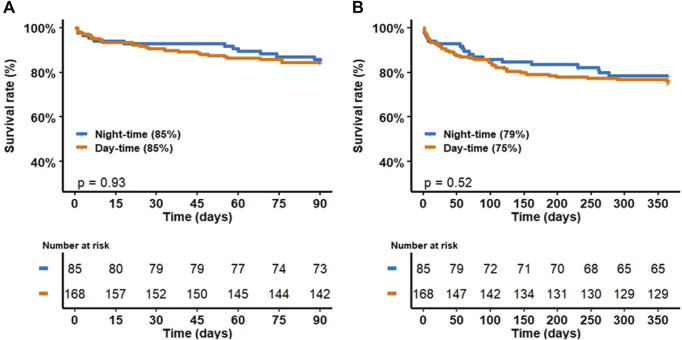
Mortality at 90 days **(A)** and 1 year **(B)** as a function of operative time.

There was no difference in mortality at 90 days and 1 year between patients transplanted during the day and those transplanted at night. Figure 1 shows mortality at 90 days and 1 year as a function of operative time.

### Airway Dehiscence

Given that airway dehiscence is more frequently associated with daytime transplantations, we explored the factors associated with the occurrence of airway dehiscence in our center. These data are presented in Table 3.

**TABLE 3:** Relationships between general characteristics, perioperative and postoperative variables and airway dehiscence during the first year after LT.

Variables	Univariate analysis				Multivariate analysis		
	Overall population (n = 253)	No airway dehiscence (n = 211)	Airway dehiscence (n = 42)	*p*-value	Odd-ratio	95% CI	*p*-value
Age, years, median [IQR]	57 [50, 62]	57 [50, 62]	58 [52, 62]	0.670			
Male sex, *n* (%)	162 (64)	129 (61)	33 (79)	0.032	2.03	[0.90–4.97]	0.102
BMI (kg/m^2^), median [IQR]	24.0 [20.0, 27.0]	24.0 [20.0, 27.0]	25.0 [23.0, 28.0]	0.066	0.14	[0.01–0.76]	0.067
Diabetes mellitus, n (%)	26 (10)	22 (10)	4 (9.5)	>0.999			
Chronic coronary disease, n (%)	10 (4.0)	9 (4.3)	1 (2.4)	>0.999			
Mean pulmonary artery pressure (mmHg), median [IQR]	25 [20, 30]	25 [21, 30]	25 [19, 35]	0.514			
Need of preoperative ECMO, n (%)	19 (7.5)	16 (7.6)	3 (7.1)	>0.999			
COPD/emphysema, n (%)	91 (36)	75 (36)	16 (38)	0.753			
Pulmonary fibrosis, n (%)	122 (48)	99 (47)	23 (55)	0.353			
Double LT, n (%)	173 (68)	142 (67)	31 (74)	0.407			
Need of peroperative ECMO, n (%)	178 (70)	145 (69)	33 (79)	0.202			
Peroperative transfusion ≥2 RBC, n (%)	122 (48)	103 (49)	19 (45)	0.672			
Daytime LT, n (%)	168 (66)	132 (62)	36 (86)	0.004	4.65	[1.77–15.13]	**0.004**
length of total operative time, hours, median [IQR]	7.20 [6h30, 8h50]	7h30 [6h30, 8h50]	7h05 [6h22, 8h10]	0.393			
SOFA on ICU admission, median [IQR]	7.0 [6.0, 9.0]	7.0 [6.0, 9.0]	7.5 [6.0, 9.75]	0.278			
Duration of vasopressor agent administration, days, median [IQR]	2.0 [1.0, 4.0]	1.0 [1.0, 3.0]	3.0 [2.0, 9.5]	<0.001	1.10	[1.03–1.19]	**0.007**
Need of ECMO support during ICU stay, n (%)	73 (29)	56 (27)	17 (40)	0.069			
Stage III PGD, n (%)	48 (19)	33 (16)	15 (36)	0.002			
Acute kidney injury, KDIGO stage 3, n (%)	36 (14)	29 (14)	7 (17)	0.621			

Continuous variables are expressed as medians and interquartile ranges (IQRs) and were compared using the Mann‒Whitney *U* test. Categorical variables are expressed as n (%) and were compared with Fisher’s exact test. BMI, body mass index; ECMO, extracorporeal membrane oxygenation; KDIGO, kidney disease improving global outcomes; MV, mechanical ventilation; PGD, primary graft dysfunction; LT, lung transplantation; SOFA, sepsis-related organ failure assessment; ICU, intensive care unit. The bold values are statistically significant.

## Discussion

We showed in this study that LT schedules had no influence on patient mortality at 90 days or 1 year. Nevertheless, patients who underwent transplantation during the day had a greater incidence of airway dehiscence. According to our multivariate analysis, day-time LT and prolonged administration of vasopressors were associated with increased airway dehiscence.

Given that quality of life at work for caregivers is a crucial issue, the aim of this study was to complete the analysis and determine whether night work had an influence on patient outcomes in our center. Unexpectedly, we found no difference in mortality between patients who underwent surgery during the day and those who underwent surgery at night. George et al. demonstrated that night-time transplantation had no influence on prognosis in a large retrospective study [10]. However, these North American data are difficult to translate to France, where, for example, the working hours of anesthetists and surgeons are limited to 24 h in a row, compared with 12 h in most Anglo-Saxon countries. Despite these differences, our study showed no difference. As the outcome of patients after LT is associated with a wide variety of factors [9, 17, 18], the variable operating time, which is a determinant of caregiver fatigue and concentration, did not seem to play a predominant role in our study. Nevertheless, to our knowledge, no high-powered study has taken into account many factors, including donor data, recipient data, preoperative variables and both early and late postoperative variables. The absence of any difference in mortality according to the time of the procedure was confirmed in a meta-analysis grouping together different types of transplantation, but given the heterogeneity of the transplantation and the different definitions used to define night or day work, the authors concluded that it was impossible to reach an objective conclusion [19].

Interestingly, our study pinpointed an important complication for patient outcome, airway dehiscence. Even though the associated factors are poorly described and have a rather complex pathophysiology, our study seems to show that, in our center, the occurrence of airway dehiscence seems more important during transplants occurring during the day. We propose several hypotheses, the first of which is a longer procedure duration with a prolonged duration of vasopressor use during day-time surgery. Second, the trend toward more pulmonary fibrosis occurring during the day may also be an explanation [20]. Nevertheless, univariate and multivariate analyses did not reveal this factor in the occurrence of airway dehiscence. Finally, the increased presence of residents and junior surgeons performing anastomoses during the day may be an explanation that deserves further analysis.

It is very reassuring to note that there is no difference in mortality between patients transplanted at night and during the day, which highlights the unfailing professionalism of the transplant team. Nevertheless, although little studied in the transplant context, burnout among professionals is a reality that can directly affect both the physical and mental health of the professional [21–23]. At a time when there is a shortage of healthcare professionals and growing awareness of the importance of quality of life at work, it seems important to limit night work and try to transplant more during the day. This would also make it possible to better plan the relief of surgeons and anaesthetists, and to concentrate caregivers of all transplant staff during the day for greater efficiency and to reduce burnout. To achieve this, there are a certain number of ways of improvement to recommend: In the case of donors after brain death, it seems reasonable to optimize organ removal schedules as much as possible so as to be able to transplant during the day. This strategy is increasingly used in transplant centers. *Ex-vivo* lung perfusion (EVLP) is a strategy that is also increasingly used worldwide to both increase the graft pool and optimize grafts prior to transplantation [24]. Although this strategy still needs a great deal of evaluation, EVLP could reasonably be used to optimize grafts at night so that they can be transplanted during the day. Centralized organ recovery and reconditioning centers are already implemented in some countries, and reducing the number of nighttime transplants is already one of the objectives of these structures [25]. Even if it sounds seductive, it deserves further investigation.

Our work has several limitations. First, it was a single-center study with a small sample size. Second, the choice of schedules (6–6) was chosen purely locally. Considering the pulmonary artery unclamping schedule could be a more rational choice. Third, a crude analysis of schedules without stratifying according to surgeon or anesthesiologist may lead to bias. However, the analysis based on the operator does not seem ethical. Fourth, more LT in COPD/emphysema patients were performed at nighttime, which is a source of bias because patients with fibrosis are often more fragile, and surgery in these specific patients is classically more difficult, which can have repercussions on the post-operative period. In our cohort, the longer operative time during the day and the higher proportion of dehiscence may potentially be secondary to this selection bias. Unfortunately, we do not have a rational explanation to highlight these population differences between daytime and nighttime. Finally, a longer-term analysis (i.e., 5 years) could be more relevant.

Our work has several strengths. To our knowledge, this work is the first to integrate the anesthetic time before the surgical incision rather than just the surgical duration. LTs involve many stakeholders, and anesthetic care is essential and can greatly interfere with patient outcomes. The monocentric character is obviously a limitation but can also be compared to a strength because it allows local constraints to be understood in a precise manner, particularly on the choice of schedules (6–6) with considerations of change of time for nurses or even availability of operating theaters.

In conclusion, we did not observe any difference in mortality between patients who underwent transplantation at our center at night and those who underwent transplantation during the day. A French multicenter analysis seems necessary in the future. Furthermore, the criteria for unclamping could be rational in this multicenter analysis. Finally, a measure of stress or fatigue of surgeons and anesthetists could also be integrated into the analysis. A more detailed analysis of airway dehiscence seems necessary.

## Data Availability

The raw data supporting the conclusions of this article will be made available by the authors, without undue reservation.

## Footnotes

1
http://www.r-project.org/

## Investigators of the Bichat Lung Transplant Group

Gaëlle Weisenburger, Vincent Bunel, Gilles Jebrak, Armelle Marceau, Domitille Mouren, Mathilde Salpin, Charlotte Thibaut de Menonville, Sabrina Trigueiros, Lucie Genet, Alice Savary, Gwenn Frere, Agnès Abadie, Diego Ferreira, Sandrine Tissot, Hervé Mal, Tiphaine Goletto, Cendrine Godet, Enora Atchade, Sebastien Tanaka, Aurélie Snauwert, Parvine Tashk, Nathalie Zappella, Sylvain Jean-Baptiste, Brice Lortat-Jacob, Alexy Tran-Dinh, Jules Stern, Léa Copelovici Sandrine Boudinet, Marie- Charlotte Chapalain, Stéphane Andreï, Iulia Balcan, Kévin Boussion, Nils Carrara, Claire Depondt, Aurelie Gouel, Pierre Habert, Adela Harpan, Elie Kantor, Fabien Lion, Laura Soldan, Agathe Verot, Marie Yammine, Alexandra Younes, Alexandre Mignon, Mouna Ben-rehouma, Dan Longrois, Christian de Tymowski, Arnaud Roussel, Quentin Pellenc, Pierre Cerceau, Yves Castier, Pierre Mordant, Willy Sutter, Antoine Girault.
