# Supplementary material for: Is Night Surgery a Nightmare for Lung Transplantation?
Source: Transpl Int. 2024 Jul 2;37:12816. doi: 10.3389/ti.2024.12816 (PMC11250068; doi:10.3389/ti.2024.12816)
Supplement: Supplementary file 1 [file Table1.DOCX]

Supplemental Table S1: General characteristics of the donors

| **General characteristics** | **Overall population**  **(n = 253)** | **Night**  **(n = 85)** | **Day**  **(n = 168)** | **p** |
| --- | --- | --- | --- | --- |
| Age, years, median [IQR] | 52 [41-61] | 51 [40-65] | 52 [41-60] | 0.424 |
| Male sex, *n* (%) | 140 (55) | 53 (62) | 87 (52) | 0.110 |
| Cigarette use, n (%) | 91 (36) | 35 (41) | 56 (33) | 0.220 |
| PaO2/FiO2 ratio, mmHg, median [IQR] | 394 [345-455] | 374 [338-462] | 404 [349-454] | 0.327 |
| Length of MV, days, median [IQR] | 2 [1-3] | 2 [1-3] | 2 [1-3] | 0.499 |

Continuous variables are expressed as medians and interquartile ranges (IQRs) and were compared using the Mann‒Whitney U test. Categorical variables are expressed as n (%) and were compared with Fisher's exact test. MV: mechanical ventilation.
