# Supplementary figures and images for: Is Night Surgery a Nightmare for Lung Transplantation?
Source: Transpl Int. 2024 Jul 2;37:12816. doi: 10.3389/ti.2024.12816 (PMC11250068; doi:10.3389/ti.2024.12816)

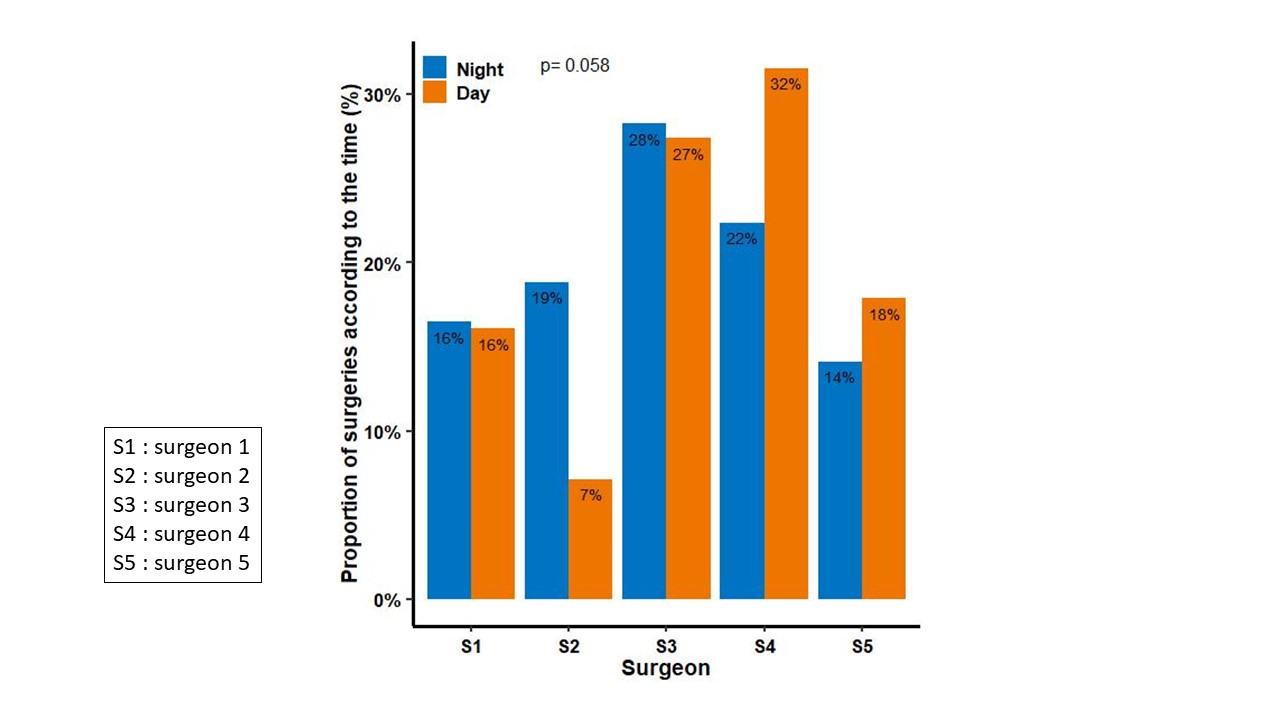

Supplement: Supplementary file 2 [file Image1.JPEG]
